# Breast Tissue Organisation and its Association with Breast Cancer Risk

**DOI:** 10.1186/s13058-017-0894-6

**Published:** 2017-09-06

**Authors:** Maya Alsheh Ali, Kamila Czene, Louise Eriksson, Per Hall, Keith Humphreys

**Affiliations:** 1 Department of Medical Epidemiology and Biostatistics, Karolinska Institutet, Stockholm, Sweden; 2 Swedish eScience Research Centre (SeRC), Karolinska Institutet, Stockholm, Sweden; 3 Department of Oncology and Pathology, Cancer Centre Karolinska, Karolinska Institutet and University Hospital, Stockholm, Sweden

**Keywords:** Mammography, Spatial organisation, Breast cancer risk, Adipose distribution

## Abstract

**Background:**

Mammographic percentage density is an established and important risk factor for breast cancer. In this paper, we investigate the role of the spatial organisation of (dense vs. fatty) regions of the breast defined from mammographic images in terms of breast cancer risk.

**Methods:**

We present a novel approach that provides a thorough description of the spatial organisation of different types of tissue in the breast. Each mammogram is first segmented into four regions (fatty, semi-fatty, semi-dense and dense tissue). The spatial relations between each pair of regions is described using so-called forces histograms (FHs) and summarised using functional principal component analysis. In our main analysis, association with case–control status is assessed using a Swedish population-based case–control study (1,170 cases and 1283 controls), for which digitised mammograms were available. We also carried out a small validation study based on digital images.

**Results:**

For our main analysis, we obtained a global *p* value of 2×10^−7^ indicating a significant association between the spatial relations of the four segmented regions and breast cancer status after adjustment for percentage density and other important breast cancer risk factors. Our (spatial relations) score had a per standard deviation odds ratio 1.29, after accounting for overfitting (percentage density had a per standard deviation odds ratio of 1.34). The spatial relations between the fatty and semi-fatty tissue and the spatial relations between the fatty and dense tissue were the most significant. The spatial relations between the fatty and semi-fatty tissue were associated with parity and age at first birth (*p*=6×10^−4^). Using digital images, we were able to verify that the same characteristics of tissue organisation can be identified and we validated the association for the spatial relations between the fatty and semi-fatty tissue.

**Conclusions:**

Our findings are consistent with the notion that fibroglandular and adipose tissue plays a role in breast cancer risk and, more specifically, they suggest that fatty tissue in the lower quadrants and the absence of density in the retromammary space, as shown in mediolateral oblique images, are protective against breast cancer.

## Background

Recent years have seen intensive efforts put into searching for relevant information from mammograms to assist the prediction of breast cancer risk. Mammographic breast density, which represents the amount of fibroglandular tissue in the breast, is the only strongly established image-based risk factor for breast cancer [1]. It is measured quantitatively either as the total dense area or the percentage of dense area on the mammogram (percentage density or PD). Women exhibiting a high PD, e.g. over 75%, have an approximately sixfold increased risk of breast cancer compared to women with a low PD (< 5%) [1]. However, the underlying mechanisms of this association are still unclear. Although PD aims at measuring the amount of dense tissue in the breast, it indirectly reflects the quantity of fat. The role of non-dense tissue in cancer development has been investigated by several researchers with contrasting outcomes [2, 3].

Mammograms present a two-dimensional projection of the breast, superimposing several layers of tissue into a single image. Hence, some researchers argue that measures of dense mammographic volume should be more accurate for classifying risk than measures of dense mammographic area [4]. These measures have still not been studied as extensively as area-based methods and Keller et al. [5] suggest that volumetric and areal density measures may be complementary for breast cancer risk assessment.

Aside from mammographic breast density, additional relevant information can be extracted from mammograms. Numerous studies have investigated the relations between cancer risk and the heterogeneity of the mammographic parenchymal pattern using quantitative texture descriptors. These particular features have been extracted on different scales, from the entire breast region [6, 7] to specific regions of interest such as the retroareolar area [8] and the central area of the breast [9].

**Fig. 1:**
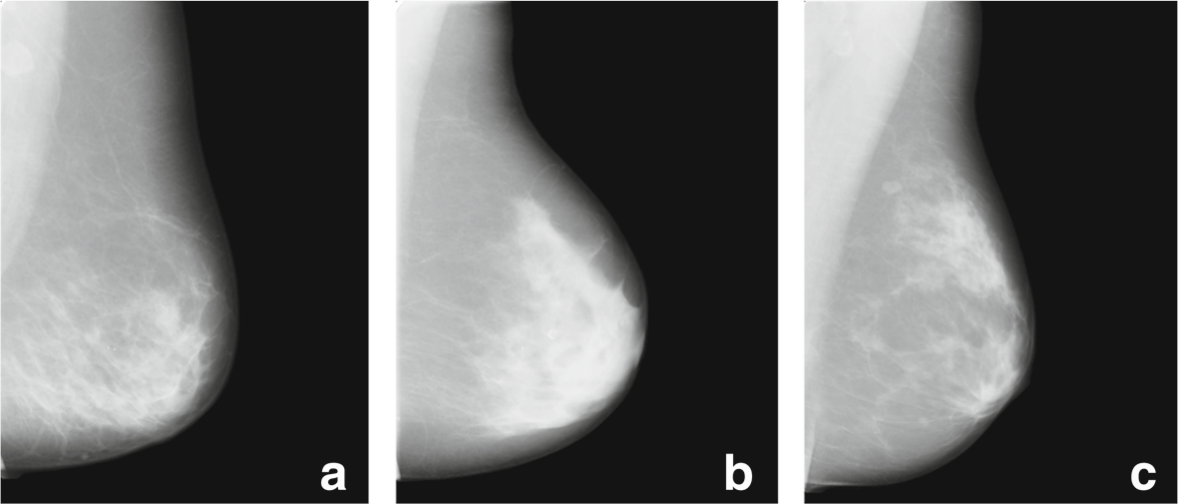
Examples of mammograms exhibiting different distributions of fatty and dense tissue. **a** The dense tissue is mainly located on the lower part of the breast. **b** The bulk of the dense tissue is concentrated in the retroareolar area. **c** The dense tissue is scattered but falls into two clusters, one next to the nipple and the other in the upper part of the breast

Another type of information present in mammograms, though seldom measured, is the spatial organisation (relations) of the different types of tissue in the breast. Whilst mammographic breast density summarises the relative amounts of dense and fatty tissue in the breast and different texture features measure the local interactions between pixel intensities, spatial relations quantitatively capture the global layout of dense and fatty tissue. It has been previously suspected that the relative distribution of adipose and fibroglandular tissue is involved in breast cancer development [10]. Figure 1 shows three different mammograms to illustrate different types of distributions of fatty and dense tissue inside the breast. Such differences could be identified by, for instance, applying basic shape descriptors on the segmented dense tissue and by measuring the distance from its centroid to the skin line [5]. However, measuring only a specific spatial relation is rarely sufficient to describe fully the possibly complex relationships between two objects. In this article, we use a novel approach that provides a more complete description of the spatial organisation of different types of tissue in the breast. We use the so-called forces histograms (FHs), which represent quantitative fuzzy spatial relation descriptors of the pairwise relations of different regions of interest. A single FH takes into account both the directional and distance relationships as well as the shapes of two objects [11]. FHs have been used in other fields of medical image analysis [12, 13] and we recently described how they can be applied to analyse mammograms [14]. In [14], we also carried out a pilot case–control study on 500 mammographic images.

In our main analysis based on digitised mammograms, we show, using 1170 cases and 1283 controls, that spatial relation FHs hold important information for discriminating between breast cancer cases and controls, after taking into account PD and other important breast cancer risk factors. We present additional analyses that shed light on the biological information captured by the FHs that are most strongly associated with breast cancer risk. We also present a small validation study based on 300 digital mammograms (69 cases and 231 controls).

## Methods

### Materials

Our main analysis is based on CAHRES (Cancer and Hormone Replacement Study), a population-based post-menopausal breast cancer case–control study of Swedish residents born in Sweden and aged 50 to 74 years, between 1 October 1993 and 31 March 1995. The study includes approximately 6000 women (3000 cases and 3000 age group matched controls) from which area-based PD measurements of digitised mammograms are available [15]. Body mass index (BMI) was recorded at entrance to the study, whereas age, in this study, was assessed according to date of mammography. All cases had primary invasive breast cancer. We used the mediolateral oblique (MLO) view since it offers the best opportunity to visualise the maximum amount of breast tissue in a single image. Mammograms were digitised with an Array 2905HD Laser Film Digitizer.^1^ Density resolution was set at 12 bits, spatial resolution at 5.0 mm and optical density at 0–4.7. The size of the images was 4770 × 3580 pixels with 0.05 mm per pixel. For the present study, we used 2453 mammograms (1170 cases and 1283 controls) for which data on PD, age, BMI, hormone replacement therapy (HRT) status, parity and age at first birth (AFB) were available for all women included. Of these, 500 (250 cases and 250 controls) were previously included in our pilot study [14].

We carried out a small validation study using digital mammograms from the Karolinska Mammography cohort (KARMA) study (http://karmastudy.org/), which is a prospective cohort study that was initiated in January 2011. Recruitment ended in March 2013. It comprises women attending mammography screening or clinical mammography at four hospitals in Sweden [16]. Participants answered a comprehensive web-based questionnaire, allowed storage of mammograms and accepted linkage to national breast cancer registers. Identification of KARMA participants as cases or controls for the present study was based on linkage with the Swedish Cancer Registry (last updated 31 December 2013). Here, we included 69 incident cases (59 with primary invasive cancer and 10 with ductal carcinoma in situ) with full field digital mammography images (raw MLO images) from GE Medical Systems, model Senographe Essential version ADS 53.40. We selected an additional 231 healthy controls with images taken with the same machine (so that, in total, our validation study was based on 300 post-menopausal women). The size of the images was 3062 × 2394 pixels with 0.1 mm per pixel. Information on age, BMI, HRT status and reproductive history was collected via a web-based questionnaire at study entry.

In both studies, for cases, all mammograms were taken less than 3 years before diagnosis (and at latest, at date of diagnosis). For cases, we used the mammogram contralateral to the tumour to ensure that image measurements were not affected by the tumours, whereas for controls an image of a single side was selected at random. This is common practice in case–control studies using mammographic images [17, 18]. For KARMA controls, all mammograms were taken at questionnaire date and, therefore, had a confirmed negative follow-up of at least 9 months. For CAHRES, mammograms of the controls were taken within 3 years of questionnaire date (the majority of mammograms were from before questionnaire date, but some were from several months after) and were all from before the diagnosis date of the last incident case, so that they were free from a breast cancer diagnosis.

### Percentage density measurement used in this study

For CAHRES, an area-based PD for each image was measured using the user-assisted approach of Cumulus [19]. Cumulus is the most widely used software for measurement of mammographic density in analogue images. The user first needs to trace and remove the pectoral muscle manually. Then, they use sliders to perform global (breast region) and then local (dense region) interactive thresholding. A trained user (LE) carried out the Cumulus measurements blinded to case–control status. KARMA images were not read by Cumulus. For these images, we used an automated measure of area PD, which has been shown to perform in line with other established density measures in terms of breast cancer risk association [20].

### Spatial relations measurements

#### Image preprocessing

The main aim of the preprocessing step is to separate the breast from other objects in the mammogram (i.e. labels, tags and screening artefacts) with a minimum loss of breast tissue. In general, two independent steps are performed. The first aims to segment the breast region, while the second separates the pectoral muscle from the rest of the breast area. The pectoral muscles were removed from both digital and digitised images using the texture gradient-based approach proposed by Bora et al. [21].

Digitised mammograms were rescaled to have pixel values between 0 and 1 and denoised using pixelwise adaptive Wiener filtering. The strong signal-to-noise ratio of the digital images allowed us to segment the breast region by applying a simple thresholding to the image. To segment the breast profile, the contrast of the image first needed to be enhanced to brighten the low-intensity pixels close to the skin line. This was achieved using a logarithmic transformation of the image. The image was then segmented into three classes according to Otsu’s multi-thresholding method. The two brightest classes were kept since they correspond to the breast region and the other objects in the mammogram, while the darkest corresponds to the background. The breast mask was finally extracted as the largest group of connected components and smoothed using morphological filtering.

#### Partition of breast images

After breast region segmentation, pectoral muscle removal and contrast enhancement for raw digital images, breast regions representing different types of tissue need to be defined. In the literature, there is no standard for the number of regions that can be defined from an X-ray image of the breast, and the number used has varied from two to 13 [22], often depending on the purpose of the study. In our work, we defined four different regions in the breast. The choice of four regions is common in the literature and corresponds to the number of original parenchymal patterns defined by Wolfe [23], who categorised images according to both the extent of densities and their characteristics (prominence of ducts and dysplasia). Also, four regions representing very dense tissue (both fibrotic stromal and glandular tissue), the fatty background of the mammogram and the fatty breast edge have been used for extracting textural descriptions [24]. Here, we used a fully automated segmentation method, the fuzzy C-Means clustering, to divide the breast into four regions loosely representing the fatty, semi-fatty, semi-dense and dense tissue.

#### Forces histogram

The FH method is applied to describe comprehensively the relative positions of the different regions of tissue. Since the FH encapsulates in a single histogram the directional and the distance relationships between the regions considered as well as their shapes, we construct an exhaustive description of the spatial organisation of tissue in a mammogram by computing the FH between all pairs of regions.

**Fig. 2:**
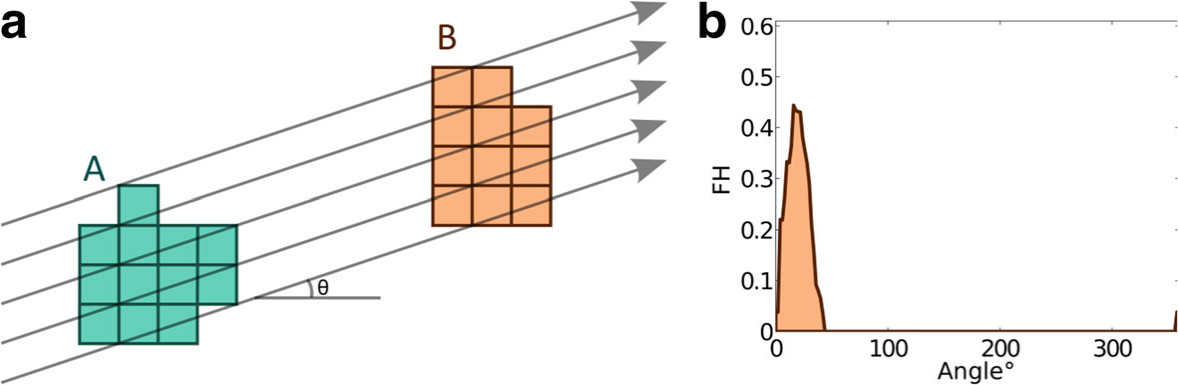
Illustration of the computation of FHs between two objects A (green) and B (orange). **a** Both objects and the parallel lines sweeping across the image oriented by a specific angle *θ*°. **b** The final FH (with angles between 0° and 360°) describing the spatial relationships between A and B. In this example, the maximum value of the FH is obtained for an angle of approximately 18° and is empty for values between 50° and 350°, since no line meets both objects A and B along these orientations. FH forces histogram

Figure 2 illustrates the principle of the computation of the FH between two sets of pixels, in this example comparing pixels within a region A (in green) with pixels within a region B (in orange). The value of the FH for a specific angle *θ* is obtained using the following algorithm. First, a series of parallel lines sweeping across the image in direction *θ* is defined. Then, for each line, a weight is calculated as the sum of the inverted squared Euclidean distances between all pairs of pixels, such that the first pixel belongs to object A and the other to B. Finally, the weights of all the lines are summed to generate the value of the FH along this particular direction, which corresponds to a single bin of the histogram. This procedure is repeated for a series of angles evenly distributed between 0° and 360°. The number of angles considered defines the length of the histogram and its angular precision, hence shorter descriptions are less accurate.

**Fig. 3:**
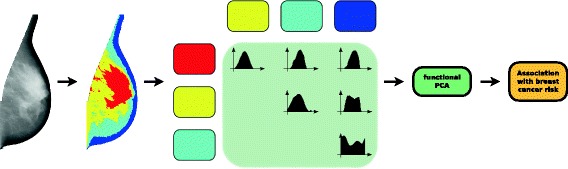
Overview of main analyses. A given breast image is first segmented into four regions: dense (red), semi-dense (yellow), semi-fatty (light blue) and fatty (dark blue) tissue. The spatial relations between each pair of regions is described by a forces histogram. The information captured by each forces histogram is compressed into a small number of variables using a functional principal component analysis and then the association between these variables (representing the spatial organisation of the regions) and breast cancer status is evaluated through a statistical test. PCA principal component analysis

Each image of the breast, segmented into four regions, is then described by a set of six FHs (4 choose 2) measuring the relative positions of the different pairs of regions. We use FH_*ij*_ to denote the FH that measures the relative positions of regions *i* and *j*. FH descriptions for an example mammographic image are shown as part of Fig. 3 (which is an overview of the complete strategy for our main analysis, which we continue to describe in ‘Statistical methods’). The computation of the FH follows a complexity of $\mathcal {O}(an\sqrt {n})$, where *n* is the number of pixels in the image and *a* is the number of angles considered. To reduce the computation time while retaining the overall spatial organisation of the tissue, each image was rescaled to 0.25 mm per pixel and we set the number of angles to 180 (a step of 2°). For more details, see [14].

### Statistical methods

Prior to testing for association with breast cancer status, for each study, we compressed the key information gathered in our six FHs (described by, in total, 6×180 FH variables) into a small number of variables. Each FH can be viewed as a function of the angle *θ*. To summarise the information contained in each FH, we apply approaches that are suitable for analysing data. These provide information about phenomena varying over a continuum (i.e. curves). Specifically, we chose to extract the dominant modes of variations included in each FH by carrying out a functional principal component (fPC) analysis [25]. We used the method described in [26], which is implemented in PACE,^2^ a MATLAB package for functional data analysis. This approach transforms the FH data to *K*-dimensional multivariate data consisting of the first *K*(fPC) scores, which account for a cumulative variance of 85%. In CAHRES, for all FHs, we retained two fPCs per FH except for the one relating regions 2 and 4, for which we retained three fPCs (13 fPCs, in total). Exactly the same number of fPCs (for all FHs) was retained when analysing the KARMA images (again 13 fPCs, in total). We will denote the *k*th fPC for the FH that measures the relative positions of regions *i* and *j* by $\text {fPC}_{ij}^{k}$. For CAHRES, we carried out our main association analysis by describing breast tissue organisation using all 13 fPC variables. This step of the analysis is represented by the box ‘functional PCA’ in Fig. 3.

We evaluated the association between the fPCs and case–control status in CAHRES by fitting logistic regression models treating case–control status as a dependent variable and the fPCs as independent variables. All fPCs were standardised to zero mean and unit variation prior to being included in logistic regression models. We included age, BMI, PD, HRT, parity and AFB (the variables parity and AFB were combined into a single categorical variable with five categories; see Table 1) in all logistic regression models as adjustment variables. In our main analysis, we chose to transform PD by taking its square root prior to including it as an adjustment variable, although we also repeated our analysis with PD on its original scale as well as categorised into five groups ([0,5[, [5,10[, [10,20[, [20,40[ and [40,100]). In our data, the overall model fit was best using square root PD. To evaluate whether our selected features (fPCs) were, overall, associated with breast cancer status (after adjustment for potential confounding variables), we carried out a likelihood ratio test. This step of the analysis is represented by the box ‘Association with breast cancer risk’ in Fig. 3.

**Table 1:** Key characteristics of individuals (CAHRES)

Characteristic	Cases	Controls	*P* value
Number	1,170	1,283	
HRT use			2×10^−9^
Never	791 (68%)	998 (78%)	
Past	98 (8%)	40 (3%)	
Current	281 (24%)	245 (19%)	
Parity and AFB			7×10^−7^
Nulliparous	157 (13%)	129 (10%)	
Parity ≤2 and AFB ≤25	349 (30%)	354 (28%)	
Parity ≤2 and AFB >25	372 (32%)	351 (27%)	
Parity >2 and AFB ≤25	214 (18%)	351 (27%)	
Parity >2 and AFB >25	78 (7%)	98 (8%)	
Age	62.6 (±6.5)	63.6 (±6.4)	8×10^−5^
BMI	25.2 (±3.6)	25.0 (±3.8)	0.26
PD	18.7 (±14.6)	14.8 (±13.2)	4×10^−12^
$\sqrt {\text {PD}}$	3.9 (±1.7)	3.5 (±1.7)	6×10^−14^

Means (with standard deviations in parentheses) are given for continuous variables and counts (with percentages in parentheses) are given for categorical variables. *P* values are obtained using likelihood ratio tests based on fitting logistic regression models without adjustment for additional covariates

*AFB* age at first birth, *BMI* body mass index, *HRT* hormone replacement therapy, *PD* percentage density

We estimated an effect size for a risk score constructed from the fPC variables of the spatial relations. If a single dataset is used naively both to train a score and to evaluate its effect size, the effect size will be overestimated (which we will refer to as an apparent estimate). We, therefore, used a bootstrapping procedure that provides a nearly unbiased (honest) estimate of the effect size (the procedure is, in fact, slightly biased in the direction of underestimating the effect); see Harrell et al. [27]. We also estimated area under the receiver operating characteristic curve (AUC) values (honest values were calculated using the bootstrap procedure) and used Delong’s test [28] for comparing apparent AUC values.

After carrying out our global test of association and estimating AUC values and an effect size for a spatial relation score, to interpret our results, we identified the most important fPCs using a stepwise selection procedure (using the step function in R). Two fPCs were selected. For these two fPCs, to identify the angle(s) (in their corresponding FHs) for which they predominantly capture variability, we visualised plots of modes of variations [29] and eigenfunctions (see Figure 6 in the Appendix). To understand the fPCs better, we studied their association with risk factors considered in our case–control analysis. Since (both) selected fPCs were approximately normally distributed, we did this by fitting linear regression models with normal error distributions.

For our validation study (KARMA), we retained only the two fPCs that were selected in our analysis based on CAHRES data. We verified that the fPCs extracted from CAHRES and KARMA carried corresponding information (using plots of modes of variations and eigenfunctions; see Figure 7 in the Appendix). Associational analysis in KARMA was also based on logistic regression analysis.

## Results

### Main analysis

For CAHRES, key characteristics of cases and controls included in our analyses are described in Table 1. We refer to the four density regions segmented inside the breast as 1, 2, 3 and 4, corresponding to the fatty, semi-fatty, semi-dense and dense tissue, respectively. The 13 fPCs, which describe breast tissue organisation (see ‘Statistical methods’), were used as covariates in the logistic regression model to investigate the association of tissue organisation with breast cancer status.

Parameter estimates of the logistic regression with all covariates (fPCs) and adjustment variables are shown in Table 2. We obtained a global *p* value of 2×10^−7^ from a likelihood ratio test on 13 degrees of freedom, indicating significant association between the spatial relations of the four segmented regions and breast cancer status. To ensure that this association is independent of breast size (it is possible that the FHs, to some extent, capture breast size), we also adjusted for the breast mammographic area; the *p* value was then 1×10^−7^. We also noted that the global *p* value was largely unchanged when PD was on its original scale (*p*=5×10^−8^), or when it was coded as a categorical variable (*p*=7×10^−7^). For an independent validation of our pilot study result, we removed the 500 images that had been included in [14]. When doing so, we obtained a *p* value of 8×10^−5^.

**Table 2:** Logistic regression results with age, BMI, $\sqrt {\text {PD}}$, parity and AFB, HRT and fPCs as covariates (CAHRES)

Covariate	Estimated	Standard	*P* value
	coefficient	error	
Intercept	−1.828	0.667	0.006
Age	−0.008	0.007	0.259
BMI	0.066	0.015	2×10^−5^
$\sqrt {\text {PD}}$	0.199	0.040	6×10^−7^
Parity and AFB			
Nulliparous			
Parity ≤2 and AFB ≤25	−0.186	0.149	0.213
Parity ≤2 and AFB >25	−0.139	0.147	0.346
Parity >2 and AFB ≤25	−0.666	0.156	2×10^−5^
Parity >2 and AFB >25	−0.393	0.201	0.051
HRT use			
Never			
Past	0.737	0.201	2×10^−4^
Current	0.270	0.108	0.013
Spatial relations fPCs ^a^			
$\text {fPC}_{12}^{1}$	−0.150	0.130	0.250
$\text {fPC}_{12}^{2}$	−0.395	0.100	8×10^−5^
$\text {fPC}_{13}^{1}$	0.004	0.098	0.967
$\text {fPC}_{13}^{2}$	0.144	0.092	0.118
$\text {fPC}_{14}^{1}$	−0.208	0.091	0.023
$\text {fPC}_{14}^{2}$	0.039	0.066	0.554
$\text {fPC}_{23}^{1}$	0.148	0.121	0.219
$\text {fPC}_{23}^{2}$	0.097	0.091	0.283
$\text {fPC}_{24}^{1}$	−0.022	0.093	0.812
$\text {fPC}_{24}^{2}$	0.158	0.078	0.042
$\text {fPC}_{24}^{3}$	0.006	0.066	0.926
$\text {fPC}_{34}^{1}$	0.118	0.065	0.069
$\text {fPC}_{34}^{2}$	0.081	0.066	0.217

*AFB* age at first birth, *BMI* body mass index, *HRT* hormone replacement therapy, *PD* percentage density ^a^ *p*=2×10^−7^

We next constructed a spatial relations score, as a sum of the fPC values weighted by the estimated coefficients (for the fPCs), as shown in Table 2. From fitting a logistic regression model with this score (instead of the 13 fPC variables), along with PD and the other breast cancer risk factors, and multiplying the standard deviation of the score by its regression coefficient, we obtained a naive (biased) per standard deviation effect size estimate of 0.33 (the per standard deviation odds ratio was 1.39) and an apparent AUC value of 0.654. Delong’s test gave a *p* value of 6.46×10^−4^ when comparing this model to one that excluded our score (which had an apparent AUC of 0.630). Using a bootstrapping procedure (based on 1000 bootstrap samples), we obtained an honest estimate of 1.29 for the per standard deviation odds ratio for the fPC-based score, and an honest estimate of 0.637 for the AUC for the full model (the honest estimate of AUC for the model excluding our score was 0.621). We note that from fitting a model that excluded all spatial relations variables, we estimated the per standard deviation odds ratio for PD to be 1.34 (the per standard deviation odds ratio for PD actually increased, to 1.40, when the fPCs of the spatial relations were included as covariates in the model).

The main features of the fPCs have to be identified to gain insight into the biological reasons for the association of the fPCs with breast cancer status. Due to the organisation of the segmented regions, it is expected that different fPCs carry overlapping information. Based on the full model (Table 2), it appears that the fPCs describing the relative position of the different regions to the fatty breast edge (region 1) are most important. After using stepwise selection, we retained $\text {fPC}_{12}^{2}$ and $\text {fPC}_{14}^{1}$. When fitting a logistic regression model with these two variables as covariates and with all the considered confounders, the corresponding estimated coefficients for $\text {fPC}_{12}^{2}$ and $\text {fPC}_{14}^{1}$ were −0.124 with a *p* value of 0.006, and −0.201 with a *p* value of 3×10^−5^, respectively. The combined *p* value for these two fPCs was 7×10^−8^. These two fPCs were moderately correlated (*r*=0.3) and both were approximately normally distributed.

To visualise the variation in FHs between regions 1 and 2, captured by $\text {fPC}_{12}^{2}$, we plotted the second mode of variation, along with its FH data and eigenfunction; see Figure 6 (a to c) in the Appendix. From the mode of variation and the corresponding eigenfunction it can be seen that $\text {fPC}_{12}^{2}$ captures variability at angles of 54°, 152° and 268°. We note that we obtained a multiple *R* ^2^ value of 0.99 based on fitting a linear regression model to $\text {fPC}_{12}^{2}$ with FH_12_(54°), FH_12_(152°) and FH_12_(268°) included as covariates. FH_12_(54°) and FH_12_(268°) are positively associated with $\text {fPC}_{12}^{2}$ whereas FH_12_(152°) is negatively associated with $\text {fPC}_{12}^{2}$.

**Fig. 4:**
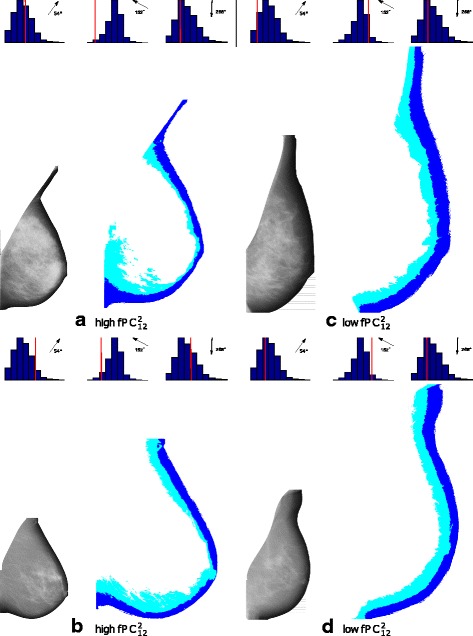
Examples of mammograms with high ((**a**) and (**b**)) and low ((**c**) and (**d**)) values of $\text {fPC}_{12}^{2}$. Original mammograms are shown next to their segmented regions 1 (dark blue) and 2 (light blue). Histograms of F*H*_12_ values at angles of 54°, 152°and 268°are included above each image with the value for the specific image marked as a vertical red line. A low $\text {fPC}_{12}^{2}$ value, after adjustment for PD and other covariates, is associated with increased risk of breast cancer

We next selected images with high and low values of $\text {fPC}_{12}^{2}$; four images are shown in Fig. 4. These images (a to d) have PD values of 53, 9, 35 and 9, and standardised $\text {fPC}_{12}^{2}$ values of 2.5, 3.0, −1.9 and −1.9, respectively. Histograms for FH_12_(54°), FH_12_(152°) and FH_12_(268°) are included above each image, with the specific FH value of an image marked as a vertical red line. From scrutinising the images and their FH values, it seems that low $\text {fPC}_{12}^{2}$ values are linked to regular distributions of both fatty and semi-fatty tissue along the skin line of the breast, whereas high $\text {fPC}_{12}^{2}$ values are related to thinning regions near the retroareolar area and irregular spreading of semi-fatty tissue in the lower part of the breast (fatty and semi-fatty tissue are collected in the lower quadrants of the images).

**Fig. 5:**
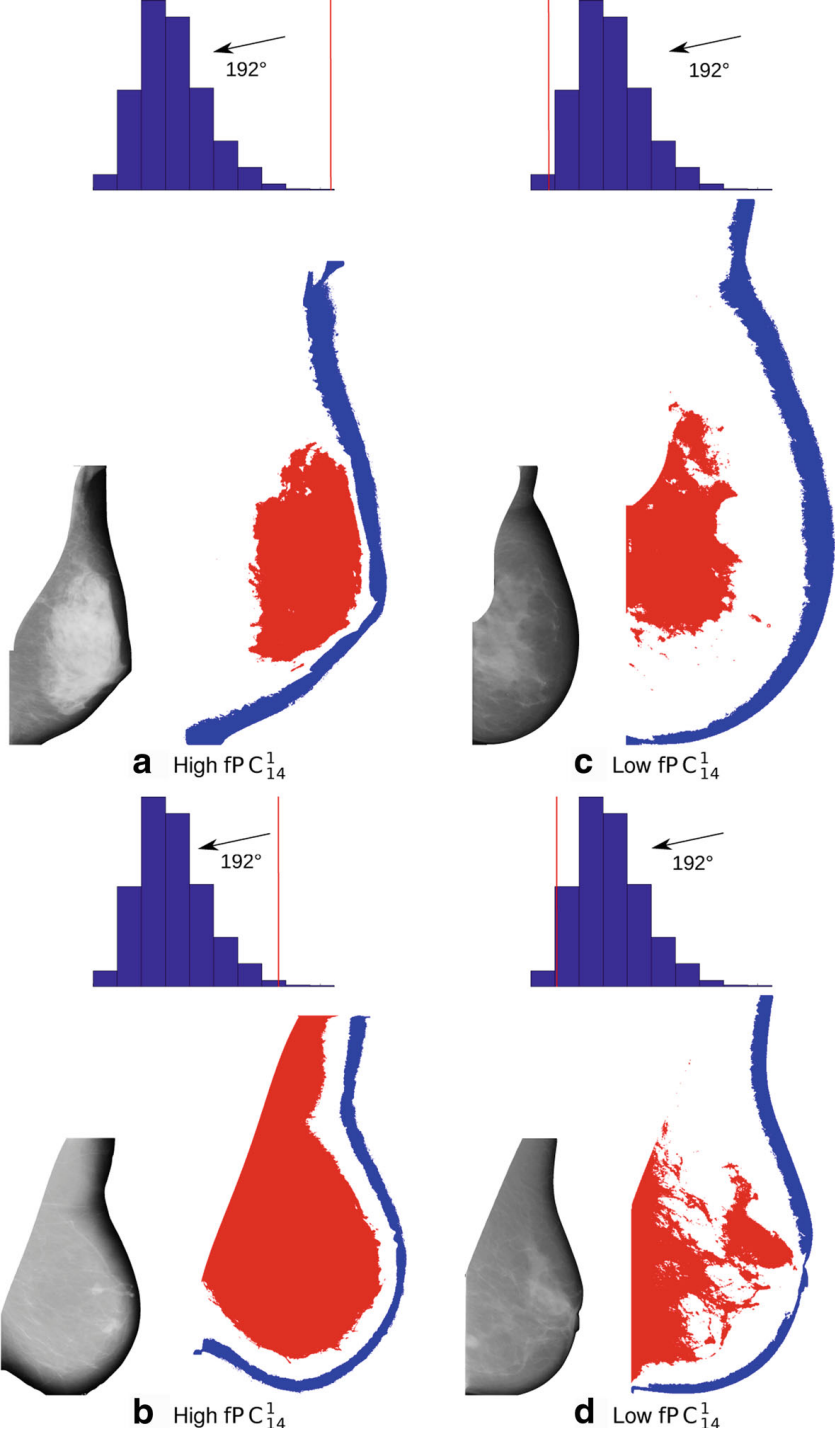
Examples of mammograms with high ((**a**) and (**b**)) and low ((**c**) and (**d**)) values of $\text {fPC}_{14}^{1}$. Original mammograms are shown next to their segmented regions 1 (dark blue) and 4 (red). The histograms of F*H*_14_ at an angle of 192° are included above each image with the value for the specific image marked as a vertical red line. A low $\text {fPC}_{14}^{1}$ value, after adjustment for PD and other covariates, is associated with increased risk of breast cancer. PD percentage density

**Fig. 6:**
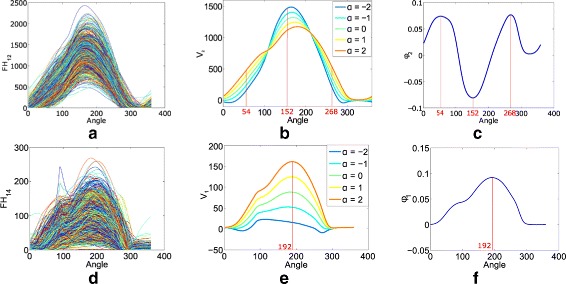
Visualisation of variability in $\text {fPC}_{12}^{2}$ and $\text {fPC}_{14}^{1}$ in CAHRES (analogue images). **a** and **d** show FH_12_ and FH_14_ for all images. The second mode of variation of FH_12_ and its eigenfunction are displayed in **b** and **c**, respectively. The red lines at angles 54°, 152° and 268° indicate the locations of the maxima of variations in the set of all the FH_12_. The first mode of variation of FH_14_ and its eigenfunction are displayed in **e** and **f**, respectively. The red line at angle 192° indicates the maximum of variation in the set of all the FH_14_

From the first mode of variation and its corresponding eigenfunction for the FHs between regions 1 and 4, it can be seen that $\text {fPC}_{14}^{1}$ captures variability at an angle of 192°; see Figure 6 (d to f) in the Appendix. FH_14_(192°) is positively associated with $\text {fPC}_{14}^{1}$ and we obtained an *R* ^2^ value of 0.93 based on fitting a linear regression model to $\text {fPC}_{14}^{1}$ with FH_4_(192°) as a covariate. Four images selected for having high or low values of $\text {fPC}_{14}^{1}$ are shown in Fig. 5. These images (a to d) have PD values of 39, 3, 19 and 3, and standardised $\text {fPC}_{14}^{1}$ values of 4.4, 3.3, −1,9 and −1.6, respectively. The value of FH_14_(192°) for each image is marked with a vertical red line on the histogram of the values of FH_14_(192°) for all images (shown above the corresponding image). $\text {fPC}_{14}^{1}$ provides information about the location of dense tissue in relation to the fatty region. From viewing images (a) and (c), two images with moderately high PD, it appears that low values of this fPC can be synonymous with the dense region being located in the retromammary space of the image (high values capture an absence of dense tissue in the retromammary space). From viewing the images (b) and (d), it may be plausible that, in low PD images, a low value of $\text {fPC}_{14}^{1}$ is synonymous with a more heterogeneous distribution of dense tissue (i.e. it is more scattered).

To trace any potential determinants of $\text {fPC}_{12}^{2}$ (key characteristics of the spatial distribution of adipose tissue) and $\text {fPC}_{14}^{1}$ (position of dense tissue relative to fatty tissue), we fitted linear regression models with, in turn, $\text {fPC}_{12}^{2}$ and $\text {fPC}_{14}^{1}$ as dependent variables, and the risk factors considered in the earlier models as independent variables. PD, parity and AFB, and BMI are all positively associated with $\text {fPC}_{12}^{2}$. Parameter estimates for the model for $\text {fPC}_{12}^{2}$ are shown in Table 3. For $\text {fPC}_{14}^{1}$, only BMI and PD have statistically significant coefficients; see Table 4.

**Table 3:** Linear regression model for $\text {fPC}_{12}^{2}$ with breast cancer risk factor covariates (CAHRES)

Covariate	Estimated	Standard	*P* value	
	coefficient	error		
Intercept	−1.332	0.272	1×10^−6^	
Age	0.003	0.003	0.373	
BMI	0.025	0.006	8×10^−6^	
PD	0.022	0.002	< 2×10^−16^	
Parity and AFB ^a^				
Nulliparous				
Parity ≤2 and AFB ≤25	0.252	0.069	2×10^−4^	
Parity ≤2 and AFB >25	0.100	0.068	0.142	
Parity >2 and AFB ≤25	0.229	0.071	0.001	
Parity >2 and AFB >25	0.108	0.093	0.243	
HRT use				
Never				
Past	−0.121	0.089	0.175	
Current	−0.074	0.050	0.140	

Pearson product–moment correlation coefficients between $\text {fPC}_{12}^{2}$ and variables age, BMI and PD are −0.04 (*p*=0.05), −0.01 (*p*=0.82) and 0.25 (*p*<2×10^−16^), respectively

*AFB* age at first birth, *BMI* body mass index, *HRT* hormone replacement therapy, *PD* percentage density ^a^ *p*=6×10^−4^

**Table 4:** Linear regression model for $\text {fPC}_{14}^{1}$ with breast cancer risk factor covariates (CAHRES)

Covariate	Estimated	Standard	*P* value
	coefficient	error	
Intercept	−2.24	0.260	< 2×10^−16^
Age	−0.003	0.003	0.224
BMI	0.078	0.005	< 2×10^−16^
PD	0.028	0.001	< 2×10^−16^
Parity and AFB			
Nulliparous			
Parity ≤2 and AFB ≤25	0.052	0.066	0.420
Parity ≤2 and AFB >25	0.086	0.065	0.188
Parity >2 and AFB ≤25	0.015	0.068	0.826
Parity >2 and AFB >25	0.004	0.089	0.959
HRT use			
Never			
Past	−0.005	0.085	0.953
Current	0.016	0.048	0.732

Pearson product–moment correlation coefficients between $\text {fPC}_{14}^{1}$ and variables age, BMI and PD are −0.10 (*p*=8×10^−7^), 0.15 (*p*=7×10^−14^) and 0.29 (*p*<2×10^−16^) respectively

*AFB* age at first birth, *BMI* body mass index, *HRT* hormone replacement therapy, *PD* percentage density

In the main association analysis (Table 2), we concentrated on looking for the main effects of fPCs. It is, of course, possible that particular features are important within narrow ranges of PD. However, when dividing the samples into three groups defined by PD ([0,7[, [7,25[ and [25,100]; the number of images per group being respectively 717, 1,176 and 560), $\text {fPC}_{12}^{2}$ and $\text {fPC}_{14}^{1}$ were jointly significantly associated with breast cancer status in all groups (with *p* values of 0.009, 8×10^−4^ and 0.006) and the signs of the coefficients were consistent across all groups.

We note that, with the exception of breastfeeding, we believe that we adjusted for the potentially most important confounders in our main analysis (Table 2). Because information on breastfeeding was missing for a substantial number of individuals (451), we did not include breastfeeding as a covariate in the main analysis. We did, however, carry out a sub-analysis. When we re-performed our analysis on a reduced data set (2002 individuals and images) with complete information on breastfeeding (yes or no), and including breastfeeding, along with the other covariates, the *p* value for association between the 13 fPCs and breast cancer risk was 2×10^−6^, which was unchanged when breastfeeding was excluded from the model. Based on fitting linear regression models with fPCs as outcome variables, breastfeeding was not significantly associated with either $\text {fPC}_{12}^{2}$ or $\text {fPC}_{14}^{1}$ after adjustment for other covariates (e.g. parity and AFB).

### Validation

A major purpose of our validation study was to check that we can identify the same characteristics of breast tissue organisation from digital images as we can from digitised analogue images. That we have a relatively small number of cases with digital images means that we have relatively low power to validate case–control associations. Key characteristics of cases and controls selected from KARMA are displayed in Table 5 in Appendix. For KARMA, as in CAHRES, 13 fPCs were retained that describe breast tissue organisation.

For further validation, we considered the two fPCs that were retained using stepwise selection in CAHRES ($\text {fPC}_{12}^{2}$ and $\text {fPC}_{14}^{1}$).

**Fig. 7:**
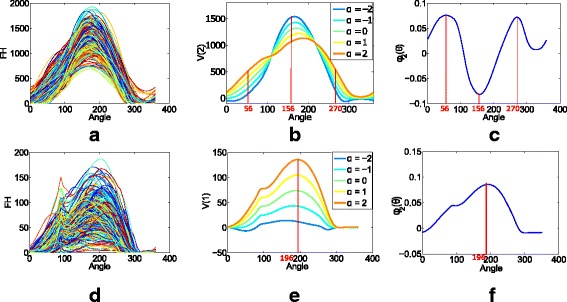
Visualisation of variability in $\text {fPC}_{12}^{2}$ and $\text {fPC}_{14}^{1}$ in KARMA (digital images). **a** and **d** show FH_12_ and FH_14_ for all images. The second mode of variation of FH_12_ and its eigenfunction are displayed in **b** and **c**, respectively. The red lines at angles 56°, 156° and 270° indicate the locations of the maxima of variations in the set of all the FH_12_. The first mode of variation of FH_14_ and its eigenfunction are displayed in **e** and **f**, respectively. The red line at angle 196° indicates the maximum of variation in the set of all the FH_14_

After constructing and examining mode of variations and eigenfunction plots (see Figure 7 in Appendix), we could confirm that these fPCs identified the same features in the KARMA digital images as they did in CAHRES analogue images. The angles capturing maximum variability in $\text {fPC}_{12}^{2}$ in KARMA were 56°, 156° and 270°, which were very close to the angles captured by $\text {fPC}_{12}^{2}$ in CAHRES. Similarly, the angle of maximum variability was almost the same over the two studies for $\text {fPC}_{14}^{1}$. When fitting a logistic regression model to case–control status, with $\text {fPC}_{12}^{2}$ and $\text {fPC}_{14}^{1}$ as covariates along with the considered confounders, the corresponding estimated coefficients for $\text {fPC}_{12}^{2}$ and $\text {fPC}_{14}^{1}$ were −0.344 with a *p* value of 0.031, and 0.243 with a *p* value of 0.128, respectively (Table 6 in Appendix). That is, despite the small number of cases, we were able to validate the association with $\text {fPC}_{12}^{2}$. This association had a *p* value that was lower than that for the association between case–control status and PD in this dataset. The apparent and honest AUCs for the model with no fPCs were 0.687 and 0.634, respectively, whilst for the model with the selected fPCs, the apparent and honest AUCs were 0.703 and 0.643, respectively. Delong’s test (based on the apparent AUCs) gave a *p* value of 0.386.

## Discussion

In this paper, we investigated the role of the spatial organisation of different regions comprising the breast, on mammograms, in terms of the risk of developing breast cancer. The results of our main analysis showed that the spatial relations between the fatty and semi-fatty tissue along with the spatial relations between the fatty and dense tissue are associated with breast cancer risk after adjustment for PD and other possible confounders. These findings are consistent with the idea that fibroglandular and adipose tissue play a role in breast cancer risk.

Several studies have already explored the role of breast fatty tissue, but have reported conflicting results [2, 3]. However, it has been noted that studies reporting a negative association between adipose tissue area and breast cancer risk based their measurements on the craniocaudal view, whereas those reporting a positive association used the mediolateral view. Our results show that the location of adipose tissue (both our fatty and semi-fatty regions) has an impact on breast cancer risk and provides additional information to explain these contrasting studies and may help in understanding the role of the fatty tissue. To define better estimates for breast cancer risk, it will likely be important to clarify the biological mechanisms regulating the spatial distribution of adipose tissue inside the breast.

In our validation study, we were able to verify that it is possible to identify the same characteristics of breast tissue organisation in digital images as in analogue images. Moreover, despite the small sample size of our validation study, we were able to validate the association between risk and our measure of the spatial relations between the fatty and semi-fatty tissue. It would, though, be valuable to study the association between the two specific FH variables identified here (or related measures developed to capture directly the important spatial features identified here) with breast cancer status using larger digital external datasets and to investigate their use in breast cancer risk prediction.

We have adopted one approach for segmenting the images prior to carrying out statistical analyses. Other approaches could be employed. Moreover, the segmentation method used in this study will always divide the breast into four regions, even for a very low-density mammogram. Since the proposed features are unavoidably influenced by the choice of the segmentation method, an adaptive approach taking into consideration the quantity of dense tissue in the breast might yield more precise descriptors, which could be easier to interpret.

Our approach could also be used on other types of images. In this study, we included only MLO mammograms. It would be interesting to investigate the spatial organisation by including craniocaudal views to give a more precise and more exhaustive description of the spatial relations of regions of density inside the breast. It would also be interesting to extend the method described here to digital breast tomosynthesis images, which provide a detailed three-dimensional view of the breast where patterns of fibroglandular tissue are not subject to overlapping tissue. Finally, we point out that the approach we described here, for capturing relevant patterns in the organisation of breast tissue, may be useful in numerous contexts, for example, in studying in detail the role of tissue density in screening sensitivity.

## Conclusions

Much attention has been paid to the dense and non-dense areas of the breast and the role of their sizes as breast cancer risk factors. In this study, we go further and find an association between the spatial organisation of breast tissue and breast cancer risk that is independent of (overall) mammographic density and a number of other established risk factors for breast cancer. The concentration of adipose tissue in the lower quadrants (which is associated with high parity and young age at first birth) and the absence of dense tissue in the retromammary space can be protective against breast cancer. These findings are completely novel and may provide a basis for more detailed biological hypotheses concerning the role of breast tissue in breast cancer.

## Endnotes

^1^ Array Corporation, Hampton, NH, USA

^2^ http://www.stat.ucdavis.edu/PACE/

## Appendix

### Modes of variation plots

For a particular fPC, of a particular FH, the set of functions (defined across a range of values of *α*) viewed on the modes of variations plots were calculated as

$$ V = \mu(\theta)\pm \alpha \sqrt{\lambda} \phi(\theta),  $$

where *μ*(*θ*) is the mean function of the considered FH, *λ* is the corresponding eigenvalue and *ϕ*(*θ*) is the corresponding eigenfunction.

### Validation study (KARMA)

**Table 5:** Key characteristics of individuals (KARMA)

Characteristic	Cases	Controls	*P* value
Number	69	231	
HRT use			0.179
Never	42 (61%)	125 (54%)	
Past	18 (26%)	86 (37%)	
Current	9 13%)	20 (9%)	
Parity and AFB			0.192
Nulliparous	9 (13%)	26 (11%)	
Parity ≤2 and AFB ≤25	19 (27%)	75 (33%)	
Parity ≤2 and AFB >25	24 (35%)	66 (29%)	
Parity >2 and AFB ≤25	15 (22%)	40 (17%)	
Parity >2 and AFB >25	2 (3%)	24 (10%)	
Age	63.06 (±6.05)	60.95 (±6.71)	0.021
BMI	25.93 (±4.48)	25.37 (±3.65)	0.283
PD	20.156 (±7.268)	18.908 (±8.252)	0.258
${\sqrt {\text {PD}}}$	4.410 (±0.8481)	4.238 (±.976)	0.187

Means (with standard deviations in parentheses) are given for continuous variables and counts (with percentages in parentheses) are given for categorical variables. *P* values are obtained using likelihood ratio tests based on fitting logistic regression models without adjustment for additional covariates

*AFB* age at first birth, *BMI* body mass index, *HRT* hormone replacement therapy, *PD* percentage density

**Table 6:** Logistic regression results with age, BMI, $\sqrt {\text {PD}}$, Parity and AFB, HRT and fPCs as covariates (KARMA)

Covariate	Estimated	Standard	*P* value
	coefficient	error	
Intercept	−8.680	2.300	2×10^−04^
Age	0.072	0.024	0.002
BMI	0.070	0.046	0.128
$\sqrt {\text {PD}}$	0.388	0.186	0.037
Parity and AFB			
Nulliparous			
Parity ≤2 and AFB ≤25	−0.490	0.502	0.330
Parity ≤2 and AFB >25	−0.022	0.485	0.963
Parity >2 and AFB ≤25	−0.125	0.528	0.816
Parity >2 and AFB >25	−1.331	0.857	0.121
HRT use			
Never			
Past	−0.772	0.347	0.026
Current	0.246	0.476	0.605
Spatial relations fPCs			
$\text {fPC}_{12}^{2}$	−0.344	0.160	0.031
$\text {fPC}_{14}^{1}$	0.243	0.160	0.128

*AFB* age at first birth, *BMI* body mass index, *fPC* Functional principal component, *HRT* hormone replacement therapy, *PD* percentage density

## Abbreviations

AFB: Age at first birth
AUC: Area under the receiver operating characteristic curve
BMI: Body mass index
FH: Forces histogram
FH_*xy*_(*θ*): Forces histogram between regions *x* and *y* at angle *𝜃*
fPC: Functional principal component
$ ext {fPC}_{xy}^{z}$: Functional principal component number *z* from the forces histogram between regions *x* and *y*
HRT: Hormone replacement therapy
MLO: Mediolateral oblique
PCA: Principal component analysis
PD: Percentage density
